# A Bayesian Density Model Based Radio Signal Fingerprinting Positioning Method for Enhanced Usability

**DOI:** 10.3390/s18114063

**Published:** 2018-11-21

**Authors:** Zheng Li, Jingbin Liu, Fan Yang, Xiaoguang Niu, Leilei Li, Zemin Wang, Ruizhi Chen

**Affiliations:** 1 State Key Laboratory of Information Engineering in Surveying, Mapping and Remote Sensing, Wuhan University, Wuhan 430079, China; 2016206440005@whu.edu.cn (Z.L.); fanyang1990@foxmail.com (F.Y.); ruizhi.chen@whu.edu.cn (R.C.); 2 Chinese Antarctic Center of Surveying and Mapping, Wuhan University, Wuhan 430079, China; zmwang@whu.edu.cn; 3 Collaborative Innovation Center of Geospatial Technology, Wuhan University, Wuhan 430079, China; xgniu@whu.edu.cn; 4 School of Computer Science, Wuhan University, Wuhan 430072, China; 5 College of Aerospace Engineering, Chongqing University, Chongqing, China, 400044; lill@cqu.edu.cn

**Keywords:** indoor positioning, fingerprinting positioning, received signal strength indication (RSSI), Bayesian density model

## Abstract

Indoor navigation and location-based services increasingly show promising marketing prospects. Indoor positioning based on Wi-Fi radio signal has been studied for more than a decade because Wi-Fi, a signal of opportunity without extra cost, is extensively deployed for internet connections. Bayesian fingerprinting positioning, a classical Wi-Fi-based indoor positioning method, consists of two phases: radio map learning and position inference. Thus far, the application of Bayesian fingerprinting positioning is limited due to its poor usability; radio map learning requires an adequate number of received signal strength indication (RSSI) observables at each reference point, long-term fieldwork, and high development and maintenance costs. In this paper, based on a statistical analysis of actual RSSI observables, a Weibull–Bayesian density model is proposed to represent the probability density of Wi-Fi RSSI observables. The Weibull model, which is parameterized with three parameters that can be calculated with fewer samples, can calculate the probability density with a higher accuracy than the traditional histogram method. Furthermore, the parameterized Weibull model can simplify the radio map by storing only three parameters that can restore the whole probability density, i.e., it is not necessary to store the probability distribution based on traditionally separated RSSI bins. Bayesian positioning inference is performed in the positioning phase using probability density rather than the traditional probability distribution of predefined RSSI bins. The proposed method was implemented on an Android smartphone, and the performance was evaluated in different indoor environments. Results revealed that the proposed method enhanced the usability of Wi-Fi Bayesian fingerprinting positioning by requiring fewer RSSI observables and improved the positioning accuracy by 19–32% in different building environments compared with the classic histogram-based method, even when more samples were used.

## 1. Introduction

Because of their social and commercial value, indoor location-based services (ILBS), which are predicted to be worth US$10 billion by 2020 and US$58 billion by 2023 [1,2], have attracted substantial attention in recent years. Smartphones, which are equipped with a variety of sensors that can be used for indoor positioning, are the most preferred platforms for such services. Techniques that are used to collect data with various sensors in a smartphone include wireless communication technologies (Wi-Fi [3], BLE [4], RFID [5,6]); optical and vision [7], and magnetic [8] among others. Among the growing number of techniques, Wi-Fi-based indoor positioning has particularly become a research hotspot [9] because Wi-Fi access points (APs) are widely deployed throughout indoor environments, such as offices and airports. As a result, Wi-Fi signals can be used for positioning signals of opportunity, thereby requiring no extra cost. In general, Wi-Fi-based indoor positioning applied in smartphone have many favorable features, such as low deployment costs, required accuracy, tolerable uncertainty, and fewer necessary computational resources [10].

Accordingly, many studies have developed different Wi-Fi-based indoor positioning approaches [11,12], such as those based on measurements of the time difference of arrival (TDOA), direction of arrival (DOA), phase of arrival (POA), and time of arrival (TOA) [12]. However, these techniques require particular hardware, which are not available with a smartphone, to acquire the corresponding measurements. Moreover, these methods need to know the locations of the APs, which are typically difficult to obtain when using signals of opportunity. As a consequence of these issues, these approaches are characterized by poor scalability. As an alternative, given a set of APs located within a space, smartphones can freely observe received signal strength indication (RSSI) measurements of Wi-Fi signals. These measurements have been used for positioning in two approaches: triangulation and fingerprinting [11]. Triangulation positioning converts RSSI measurements into distances between APs and a smartphone using a signal propagation model; therefore, it needs to know the locations of at least three APs. The performance of this approach depends on the accuracy of RSSI-based distance observables and the availability of the locations of APs [13]. A number of studies on the signal propagation model have been performed. For example, the rain attenuation effect has been incorporated into the signal propagation model [14]. Previous researches have also presented deep analysis on the impact of different disturbing phenomena such as reflections, diffraction, and scattering on the measurement accuracy [15,16]. In addition, some simple formulas to estimate the achievable accuracy have been introduced to clarify the effect of distance, antenna pattern, propagation model, and propagation conditions on the location accuracy [17]. Varying environments, APs, and smartphone hardware may cause different signal propagation model relationships between RSSI measurements and distances; hence, the accuracy and stability of triangulation positioning are limited by environmental interference of Wi-Fi signals.

In contrast, fingerprinting positioning exploits the signature of environmental variations in RSSI observables as effective measurements and therefore does not need to know the locations of the APs [18,19]. Hence, Wi-Fi fingerprinting with RSSI observables is preferable for indoor positioning. The fingerprinting algorithm consists of two phases. The first phase is radio map learning [18], which aims to establish the RSSI statistic and location relation database within the area of interest. The second phase is position inference [20], which estimates the position of the smartphone by matching the real-time RSSI measurements received by the smartphone with a radio map. In the fingerprinting approach, the efficiency and quality of radio map learning are fundamental to achieving a good positioning performance [21]. If an insufficient number of RSSI samples is utilized for radio map learning, the RSSI statistics will not be accurate, and the fingerprinting positioning accuracy will correspondingly be degraded. In the Bayesian fingerprinting approach [22,23], to enhance the radio map learning quality, as many RSSI samples as possible should be acquired to calculate the RSSI statistics. However, the acquisition of RSSI data necessitates laborious fieldwork, complex computational resources, and increased cost of both deployment and maintenance of related services. Therefore, for large-scale applications, the fingerprinting positioning approach requires an enhanced usability, which demands a suitable positioning accuracy using acceptable field acquisition, computational resources, and high-quality radio map learning costs. Some studies have introduced methods to reduce computational costs at the operational stage, such as a cluster algorithm using the coarse localization algorithm [24] and a novel metric, called the penalized logarithmic Gaussian distance metric, which can boost the performance of the clustering [25]. In another report, the fingerprint method used a previously stored map of signal strength at several positions and positioning using similarity functions and majority rules [26] to reduce additional efforts. In addition, a number of studies on radio map have been performed to reduce costs needed for radio map learning. For example, a novel method based on the radio propagation model was used to construct a radio map with full fingerprints [27]. Another research proposed RSSI measurements in some positions, with the rest of the fingerprints to be calculated by linear interpolation or Delaunay algorithm [28]. Gaussian processes regression (GPR) has also been utilized to construct the radio map [29,30]. However, these methods come at the cost of lower accuracy.

Based on the above discussion, this study proposes a Bayesian density model to represent the probability density of Wi-Fi RSSI samples of a specific location. The proposed Bayesian density model is based on the Weibull function, which contains three parameters. With the proposed model, the Bayesian fingerprinting approach is enhanced in three aspects. First, the three parameters of the Weibull function can be estimated with a limited number of RSSI samples, and the resulting Weibull–Bayesian density model can represent the probability density with a much higher accuracy than can be achieved by classic Bayesian fingerprinting positioning methods, such as the histogram [31] or kernel methods [32,33]. Thus, the amount of fieldwork and the cost of radio map learning are both reduced, and the data collection efficiency is improved. Second, the structure and computational complexity of the radio map database are simplified because the proposed method needs to store only the abovementioned three parameters of the Weibull function that can restore the complete probability density; in other words, it is not necessary to store probability distribution based on separated RSSI bins as in traditional approaches. Third, for the position inference phase, this study proposes a Bayesian inference algorithm that utilizes the Weibull–Bayesian density model. Unlike traditional methods that calculate the probability distribution of RSSI bins that are predefined, the proposed method calculates the posterior probability using the Bayesian density model and a run-time dynamically defined bin according to the real-time RSSI measurements. Then, the position solution is determined with the maximum likelihood estimation. Experiments conducted with an Android smartphone showed that the proposed method needed far fewer RSSI samples for radio map learning than traditional Bayesian positioning methods and could achieve better positioning accuracy. In this respect, the usability of the Bayesian fingerprinting positioning method was improved.

## 2. Fingerprinting Positioning Using Radio RSSI Measurements

Location fingerprinting is mainly based on a fingerprint database of target features for their identification, and the positioning process can be divided into two phases: radio map learning and position inference [18,34,35].

### 2.1. Radio Map Learning Phase

The first phase is the offline radio map learning phase, the main purpose of which is to establish a number of reference points in the target area. Following this, a smartphone can be used to collect the received signal characteristic parameter data (e.g., RSSI measurements) from multiple APs at each reference point. These parameters, in addition to the position coordinates of the point, form a set of data stored in the database [36], and the RSSI fingerprint database is called a radio map [37]. The fingerprints may represent average RSSIs (i.e., a deterministic approach) [37,38] or RSSI probability distributions (i.e., a probabilistic approach) [37,39]. The more accurate the generated radio map, the better is the positioning accuracy that can be achieved [13]. The efficiency and quality of the radio map are fundamental to implementing the fingerprinting algorithm.

In the conventional probabilistic approach, the RSSI probabilities of all APs received at each reference point are stored in the Bayesian fingerprinting positioning database [40,41]. In the conventional Bayesian algorithm, the probability of an RSSI measurement $X_{n}$ between a reference point $R_{i}$ and an AP $A_{m}$ can be expressed as follows:

(1) $$P\left(A_{m}X_{n}|R_{i}\right)=\frac{Y_{x_{n}}}{N_{i}}$$

where $Y_{x_{n}}$ is the number of times RSSI measurement $X_{n}$ appears in the training data set of the *i*-th reference point. Here, $N_{i}$ is the total number of training samples collected at the *i*-th reference point. The entire fingerprint database is expressed by the following equation:

(2) $$D=\left[R_{1},R_{2},\ldots ,R_{w}\right]$$

where w is the total number of reference points in the target area. In addition, to improve the computational process and weaken the RSSI measurements associated with signal strength singularities, a bin-based solution is adopted. The conventional algorithm divides the signal strength distribution into nine ranges, and each range can be regarded as a bin. In this way, the probability distribution of each AP is recorded bin-wise as a unit of the fingerprint database. During the positioning, the corresponding bin acquisition probability of each received AP is determined based on the received RSSI measurements [42]. In the conventional Bayesian algorithm, at the *i*-th reference point, the probability of an RSSI measurement falling within bin $B_{n}$ for an AP *A_m_* can be expressed as follows:

(3) $$P\left(A_{m}B_{n}|R_{i}\right)=\frac{\sum_{j\in B_{n}}Y_{x_{j}}}{N_{i}}=\frac{Y_{B_{n}}}{N_{i}}$$

where $B_{n}$ denotes the range of ($E_{n-1}$,$E_{n}$]; *n* is the number of bins; $E_{n-1}$ and $E_{n}$ are the left and right boundary values of bin $B_{n}$, respectively; and $Y_{x_{j}}$ denotes the number of times an RSSI measurement appears within the range of ($E_{n-1}$,$E_{n}$]. All RSSI measurements in bin $B_{n}$ are cumulated to count the probability. In this paper, the operation of this conventional acquisition probability value is called static bin. To improve upon the static bin operation, this paper proposes a more efficient operation method called dynamic bin, which will be introduced in detail below.

### 2.2. Position Inference Phase

The second phase is the online position inference phase. Using the smartphone that receives the same signal parameters stored at the positioning point, we use the corresponding matching algorithm to determine which fingerprint data in the fingerprint database are the most similar. Then, we use the most similar sets or groups of fingerprint data corresponding to the matching algorithm, and those data can be used to estimate the actual user location [36].

Two classic types of methods, namely, the deterministic approach and the probabilistic approach, are currently utilized [43]. The deterministic approach is relatively simple and boasts a more extensive range of current applications. Examples of this technique include the nearest neighbor (NN) algorithm, K-nearest neighbor (KNN) algorithm, and weighted K-nearest neighbor (WKNN) algorithm [11]. Most of the previous works have simply used the definition of Euclidean metric in the signal space to understand the minimum distance mapping within the RSSI signal space as the minimum distance for a physical location [11,43]. Many studies have also used alternative distance metrics, for instance, RSSI rank-based [44], threshold strategy [45], similarity functions, and majority rules [26,45]. During the online positioning phase, a smartphone receives a set of RSSI measurements and calculates the shortest distance from the RSSI measurements corresponding to the same AP within the fingerprint database [38]. Then, the NN algorithm outputs the position coordinates of the reference point with the shortest distance solution as a positioning result. The KNN algorithm finds the NN reference point of K (K > 2), representing the position coordinates of K reference points with the shortest distance, and takes the mean value to obtain the positioning result. Meanwhile, the optimization of the WKNN algorithm, which is based on the KNN algorithm, is employed to find the NN reference point of K (K > 2) and multiply the coordinates of each reference point by a weighting coefficient to obtain the positioning result [11,38].

However, because Wi-Fi signals are susceptible to interference within indoor environments, the signal space composed of Wi-Fi RSSI measurements does not exhibit a one-to-one mapping relationship with the physical location [46]. Therefore, the probabilistic approach can provide better accuracy and greater usability for Wi-Fi positioning than the deterministic approach [37,40,41]. In general, the Bayesian position estimation algorithm is superior to the WKNN algorithm [42,46]. Thus, the proposed method is based on the probabilistic Bayesian fingerprinting positioning approach.

## 3. Bayesian Position Estimation Approach Based on the Weibull Signal Model

The algorithm proposed in this paper is based on the probabilistic Bayesian position estimation method, and some improvements to existing problems in this method are proposed herein to enhance the accuracy and usability of the algorithm. In this paper, we optimize both the radio map learning phase and the position inference phase of the Bayesian probabilistic algorithm.

### 3.1. Weibull–Bayesian Density Model of Radio Signals

Bayesian inference in models employed to generate a density estimation is usually described using a mixture of the Dirichlet process [47]. These models [47,48] provide natural settings for estimating the density. Moreover, efficient fitting methods can be used to approximate various prior, posterior, and predictive distributions, thereby allowing inferences regarding a variety of practical issues [47], including smoothing and uncertainties in density estimates. Bayesian approaches using a mixture of the Dirichlet process provide theoretical bases for more traditional nonparametric methods, and hence, an appropriate Bayesian modeling framework can address various practical problems [47,48,49]. We therefore propose a Weibull–Bayesian density model for Wi-Fi signals.

Understanding the statistical characteristics of the RSSI probability distribution is fundamental [37,50] to implementing the proposed Bayesian position estimation method. A number of studies on the RSSI probability distribution have been performed. For example, a lognormal distribution [51] was used to model the RSSI. In another report, a shape-filtered empirical distribution was utilized to estimate the RSSI distribution [52]. In kernel methods, Gaussian-based kernel functions are usually employed to approximate the probability density function (PDF) of the RSSI [32,33,52]. Previous researches has found that the RSSI typically follows a non-Gaussian, left-skewed distribution [50,53]. In addition, an improved double-peak Gaussian distribution (IDGD) has been introduced to approximate the RSSI probability distribution [38]. However, although a normal distribution is often used for Wi-Fi RSSI measurements [13,19], some studies [37] have shown that this assumption is not always correct.

**Figure 1:**
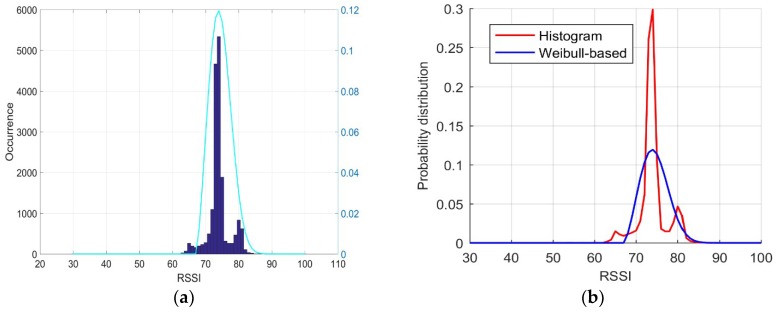
Comparison of probability distributions: (**a**) a typical comparison of the RSSI probability density derived with the histogram ($\bar{O}$ = 74, STD = 3.186) and Weibull signal model (cyan line, referring to the right axis); (**b**) Weibull-based probability distribution (k = 2.5, λ = 8.4428, *θ* = 67) with 17,874 samples (blue line) vs. the histogram probability distribution with 17,874 samples (red line). The RSSI is in the unit of –dBm in this paper.

In this study, a sampling test was performed over a period of 12 h in an indoor environment, namely, in the official building of the Central Creative Building covered with Wi-Fi signals, and more than 17,000 RSSI samples were obtained. An interesting distribution can be observed in Figure 1a,b, where the real distribution is almost entirely non-Gaussian and left-skewed. Based on this observation, in this paper, we introduce the Weibull signal model to approximate the RSSI probability distribution of all APs received at each fingerprinting point. The Weibull signal model is a traditional method for modeling the signal strength of propagation radio waves [54]. The corresponding PDF can be expressed as follows:

(4) $$\mathrm{pdf}\left(x;\lambda ,k,\theta \right)=\frac{k}{\lambda }{\left(\frac{x-\theta }{\lambda }\right)}^{k-1}e^{-{\left(\frac{x-\theta }{\lambda }\right)}^{k}}$$

Accordingly, the cumulative distribution function (CDF) can be expressed as follows:

(5) $$F\left(x\right)=1-e^{-{\left(\frac{x-\theta }{\lambda }\right)}^{k}}$$

where x is the variable of the function; k is the shape parameter; λ is the scale parameter; and θ is the shift parameter [31,42].

The parameters of the Weibull signal model can be estimated with a limited number of sampled RSSI measurements. The model parameters (λ, k, and θ) can be calculated with the following equation [53,55]:

(6) $$k=\frac{\mathrm{STD}}{\ln\left(2\right)}\;,\;1.5\leq k\leq 2.5$$

(7) $$\lambda =\{\begin{array}{cc} 2*\left(k+0.15\right) & STD<2\\ STD*\left(k+0.15\right) & 2\leq STD\leq 3.5\;\;\\ 3.5*\left(k+0.15\right) & STD>3.5 \end{array}$$

(8) $$\theta =\bar{O}-\lambda *\Gamma \left(1+\frac{1}{k}\right)$$

(9) $$\bar{O}=\frac{1}{n}\overset{n}{\underset{i=0}{\sum }}O_{i}$$

(10) $$STD=\sqrt[{}]{\frac{1}{n}\overset{n}{\underset{i=0}{\sum }}{\left(O_{i}-\bar{O}\right)}^{2}}$$

where $\bar{O}$ is the mean value of the RSSI measurements set $O_{i}$; STD denotes the standard deviation; and Γ is the gamma function. The term (k+0.15) is an approximation of the expression $1/\sqrt{\Gamma \left(1+\frac{2}{k}\right)-\Gamma^{2}\left(1+\frac{1}{k}\right)}$ when 1.5≤k≤2.5 [31,47].

Therefore, the distribution probability of each possible RSSI measurement in the fingerprint database can be expressed as follows:

(11) P(x)=F(x+0.5)−F(x−0.5)

For a fingerprint database measured with the Weibull signal model, we can calculate the probability of occurrence of any RSSI measurement. As the RSSI measurements are integers, the probability for each bin in the fingerprint database can be generated as follows:

(12) $$P\left(x\right)=\int_{x}^{x+w}f\left(x\right)dx=F\left(x+w\right)-F\left(x\right)$$

where *w* is the width of the bin, and *x* is the RSSI value at the left boundary value of bin.

The fingerprinting method using the radio map based on the Weibull–Bayesian density model can be represented by a set of Weibull signal models that simulate the distribution of RSSI measurements. Each Weibull signal model contains three parameters (λ, k, and θ) representing the probability distribution of the RSSI measurements between an AP *Am* and a smartphone at a reference point $R_{i}$. The structure of the radio map can be greatly simplified in this case because it requires storing only three parameters of the Weibull function that can restore the complete probability density; in other words, it is not necessary to store probability distribution based on separated RSSI bins as in traditional approaches.

To evaluate the performance of the proposed approach, a fitting experiment was carried out to determinate whether the shape of the Weibull signal model derived from the same RSSI samples can approximate the reference shape derived from a multitude of RSSI measurements acquired over a long recording session. For this purpose, a test was performed over a long recording session in the official building of the Central Creative Building, and 17,874 RSSI samples were acquired. Consequently, considering that the histogram probability distribution derived from the 17,874 RSSI samples collected over a 12-h period was close to the real RSSI probability distribution, we used it as the baseline distribution for the purpose of comparison.

Next, Equations (6)–(10) were used with all of the RSSI samples to calculate the parameters of the Weibull signal mode derived from the entire set of samples as follows: shape k = 2.5, scale *λ* = 8.4428, and shift *θ* = 67. The estimated Weibull signal mode was then used to compute the PDF, shown as the cyan line in Figure 1a, which was close to the real PDF, as demonstrated in Figure 1a, where the blue colored bars represent the histogram of all RSSI samples. Using Equation (11), we obtained the Weibull-based probability distribution, as shown with the blue line in Figure 1b; the red line is the baseline distribution. Evidently, the shapes of the two lines are similar.

### 3.2. Fingerprinting Positioning Using the Weibull–Bayesian Density Model

During the position inference phase, the bins were dynamically divided based on the Weibull signal model according to the RSSI measurement of each AP received in real time. Therefore, with the same fingerprinting method, we could compare the probabilities generated from the same data set using three different algorithms: the conventional Bayesian fingerprinting histogram algorithm with static bin; the Weibull bin fingerprinting algorithm based on the Weibull–Bayesian density model with static bin; and the Weibull PDF fingerprinting algorithm based on the Weibull–Bayesian density model with dynamic bin.

The fingerprinting positioning method employed in this paper relied on Bayesian theory and the histogram maximum likelihood algorithm [39]. The principle of this method, which is also called the Bayesian probability algorithm, is to use the conditional probability model for location fingerprinting and the Bayesian inference mechanism to estimate the position of the smartphone [56]. The basic principle can be expressed as follows:

(13) $$p\left(x|y\right)=\frac{p\left(y|x\right)p\left(x\right)}{p\left(y\right)}=\frac{p(y|x)p\left(x\right)}{\sum_{x^{\prime }\in X}p(y|x^{\prime })p\left(x^{\prime }\right)}$$

where x is a reference point in the fingerprint database; y is the RSSI measurements of the AP received by the smartphone at the anchor point; p(x|y) is the probability that the anchor point is the reference point *x* when the RSSI measurement is *y*; p(y|x) is the probability that the RSSI measurement is y at the reference point x; p(x) is the probability of a reference point that usually does not consider the difference between the reference points (the default is the equal probability of all reference points); and p(y) is the RSSI measurement occurring with the full probability (the default AP is usually mutually independent). From Equation (8), when the value of p(x|y) is maximum, the probability of reference point x occurring when the RSSI value received at the anchor point is *y* also reaches a maximum. In other words, the best match with the anchor point can be used as the positioning result output. Therefore, the Bayesian probability algorithm is used to find the maximum value of p(x|y) at which x is the positioning result, and the formula can be expressed as follows:

(14) $$\hat{x}=argmax_{x}p\left(x|y\right)$$

To obtain the maximum value of p(x|y), we know that p(x) and p(y) are the same at each fingerprinting point according to the Bayesian theory formula. The maximum value of p(x|y) can be transformed to solve for the maximum value of p(y|x), which represents the probability of RSSI measurements of each AP being received at reference point x. Because each AP is independent, this method determines the probability product maximum value of the RSSI measurements of each AP, and the formula can be expressed as follows:

(15) $$\hat{x}=argmax_{x}\left(\prod_{j=1}^{n_{MAC}}p\left(y_{j}|x\right)\right)$$

where $n_{MAC}$ is the total number of AP received by the smartphone at the anchor point; $y_{j}$ represents the RSSI measurements of the *j*-th AP received by the smartphone at the anchor point; *x* is a reference point in the fingerprint database. Therefore, the conditional probability product of all APs at each reference point can be calculated, the maximum probability can be found according to the histogram maximum likelihood algorithm, and the corresponding reference point is the positioning result.

In this paper, the conventional static bin method stored the probability distribution of the RSSI measurements of each AP received at each reference point in a well-defined bin as a radio map. In contrast, the dynamic bin method proposed in this paper added ±B dBm to the RSSI measurements of each AP received during the positioning phase, thereby incorporating a dynamic bin to account for the probability in Equation (5). Accordingly, we calculated a large number of Wi-Fi APs RSSI measurements and found that most of them had a standard deviation between 2 and 3, and 95% of the measurements errors were within twice the standard deviation if the errors followed the Gaussian distribution, according to the theory of the statistics. Thus, the parameter B was given the value of twice the standard deviation. Considering the differences between different APs, an experiment was carried out to compare the positioning accuracy with ±4 dBm, ±5 dBm, and ±6 dBm to identify which would be the best as the value of B. We used the proposed algorithm to calculate the average error and root-mean-square (RMS) error of each RSSI range and plotted the error distribution graphs with ±4 dBm, ±5 dBm, and ±6 dBm in addition to the probability cumulative distribution function of the positioning error. More details about the experiment will be introduced in Section 4.1.

**Figure 2:**
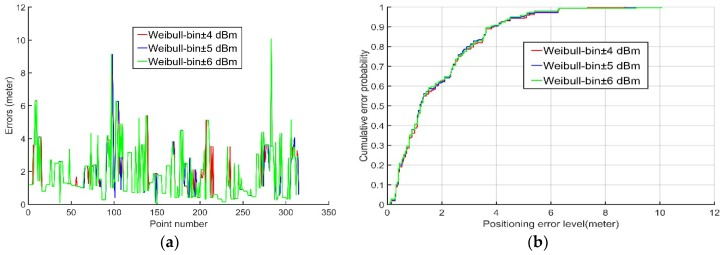
The (**a**) positioning error and (**b**) cumulative distribution function (CDFs) of different RSSI ranges.

Table 1 and Figure 2 demonstrate that, although they exhibited few differences, the positioning accuracy obtained with ±5 dBm (blue line in Figure 2) were slightly superior to those obtained with both ±4 dBm (red line in Figure 2) and ±6 dBm (green line in Figure 2). Therefore, ±5 dBm proved to be the best B value. The dynamic bin probability of RSSI measurements of each AP received at the anchor point can be expressed as follows:

(16) $$P\left(x\right)=\int_{x-B}^{x+B}f\left(x\right)dx=F\left(x+B\right)-F\left(x-B\right)$$

where x is the RSSI value of AP *A_m_* received by the smartphone at reference point $R_{i}$; B is the half width of the bin; x+B and x−B are the left and right edges of the dynamic bin, respectively; and P(x) represents the probability of occurrence at point $R_{i}$ when the RSSI value of AP *Am* is x.

The three parameters required by the Weibull–Bayesian density model are stored in the radio map to first model the probability distribution of the RSSI measurements between an AP *Am* and a smartphone at a reference point $R_{i}$ and then dynamically calculate the probability value of the bin in real time. This approach constitutes the fingerprinting algorithm based on the Weibull–Bayesian density model with the dynamic bin method proposed in this paper.

## 4. Experiments and Results

In this positioning experiment, the average error and root-mean-square error in the positioning results of the three algorithms were compared in different actual scenarios. The objective of the experiment was to compare the positioning performance of the two Weibull-based algorithms (Weibull bin and Weibull PDF) with that of the conventional histogram algorithm.

### 4.1. Experimental Environment and Process

**Figure 3:**
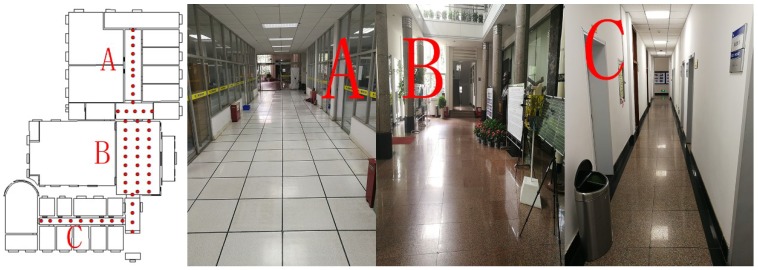
Second-floor plan: Area (**A**) is the corridor between the students’ computer labs, which is characterized by a large flow of people, and is large, and has a complex Wi-Fi signal environment. Area (**B**) is a large, spacious lobby with fewer Wi-Fi signals. Area (**C**) is the corridor between the teacher’s office characterized by a simple physical environment and a simple Wi-Fi signal environment. The area in the first picture (where there is no reference point) is private and cannot be tested.

**Figure 4:**
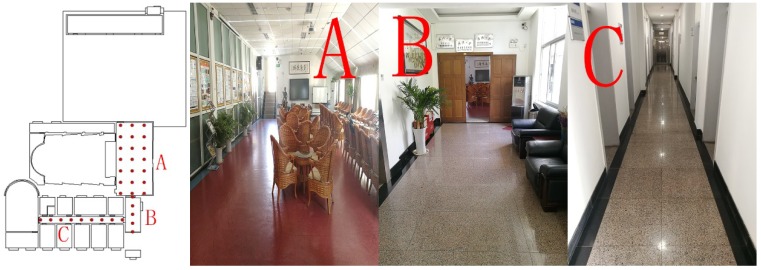
Fourth-floor plan: Area (**A**) is a large conference room that is irregular and has fewer Wi-Fi signals. Areas (**B**,**C**) are different types of corridors with simple physical environments and simple Wi-Fi signal environments. The area in the first picture (where there is no reference point) is private and cannot be tested.

In this experiment, two floor plans were selected to verify the broad applicability of the proposed algorithm in various environments. The two experiments were carried out in public areas on the second and fourth floors, both of which are 53 m by 73 m in size, within the office building of our laboratory. The second floor is mainly composed of a lobby, students’ computer labs, and multiple corridors (see the four maps in Figure 3), whereas the fourth-floor environment is sample and consists only of conference rooms and corridors (see the four maps in Figure 4). Sketches of the floor plans and actual photos are shown in Figure 3 and Figure 4.

Figure 3 and Figure 4 show that our experimental area included most actual scenarios in an office building, including a lobby and a conference room in addition to different types of corridors. In our experiment, 56 reference points were established on a grid map at intervals of 2–3 m in public areas, such as the lobby and corridors on the second floor, and 30 sets of sample data were collected at each reference point to establish the fingerprint database. Subsequently, we randomly selected 43 evaluation points within the experimental area and then measured and recorded the true coordinates of those evaluation points. At each evaluation point, five discontinuous sets of RSSI measurements were collected at different times; as a result, a total of 215 independent sets of data were collected for testing. In the same way, a total of 35 reference points were collected on the fourth floor, and 30 sets of sample data were collected at each fingerprinting point to establish a fingerprint database. A total of 20 evaluation points were selected at random, and five discontinuous sets of RSSI measurements were collected at different times at each evaluation point. In this way, a total of 100 independent sets of data were acquired for testing.

Based upon our experience, it is impractical to dedicate vast amounts of computational and human resources to acquire data at each reference point to train the database. Approximately 20 RSSI samples can be obtained over a one-minute sampling duration, and 30 RSSI samples can be obtained over a 90 s sampling duration. Therefore, we selected 20 samples as a subset of the 30 samples and 30 samples as limited sampling cases for comparison.

### 4.2. Validation of the Weibull–Bayesian Density Model

Based on the long recording session, 17,874 RSSI samples were acquired in the official building of the Central Creative Building. More fitting experiments were carried out to determine whether the shape of the Weibull signal model derived from a limited subset of RSSI samples could approximate the reference shape derived from a multitude of RSSI measurements acquired over a long recording session. The fitting experiments revealed that the model shape was very similar, even when the number of samples decreased from 10,000 to 30 or 20.

**Figure 5:**
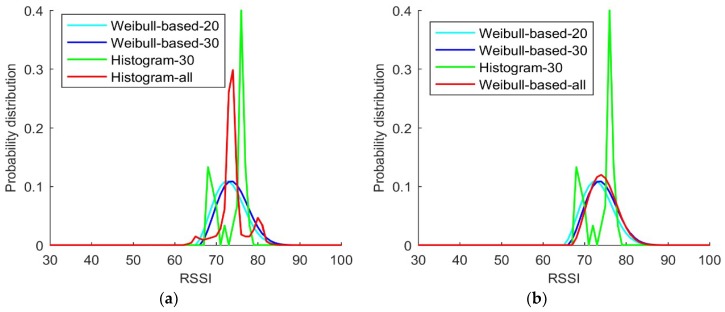
Comparison of probability distributions: (**a**) Weibull-based probability distribution with 20 samples (cyan line) vs. Weibull-based probability distribution with 30 samples (blue line) vs. histogram probability distribution with 30 samples (green line) vs. histogram probability distribution with all samples (red line). (**b**) Weibull-based probability distribution with 20 samples (cyan line) vs. Weibull-based probability distribution with 30 samples (blue line) vs. histogram probability distribution with 30 samples (green line) vs. Weibull-based probability distribution with all samples (red line).

In Figure 5a,b, the blue lines represent the probability distribution derived from a Weibull-based solution using 30 RSSI samples randomly selected from the large data set, while the cyan lines represent the probability distribution derived from a Weibull-based solution using fewer (only 20 out of 30) RSSI samples. The green lines represent the probability distribution derived from the histogram solution for the same data set of 30 RSSI measurement samples, while the red lines in the two figures are different but can be both considered as the baseline distribution according to the comparison in Figure 1. The baseline distribution is the histogram probability distribution derived from all samples in Figure 5a and the probability distributions derived from the Weibull-based solution with all samples in Figure 5b.

Figure 5a,b shows that the shape of the Weibull signal model derived from either 20 or 30 RSSI samples are similar to that of the two baseline distributions. By comparing the probabilities estimated using the conventional Histogram solution with 30 samples with those estimated using the Weibull signal model with 30 samples, it is obvious that the probabilities estimated using the latter are closer to the baseline distribution than is the former. A comparison of the probabilities estimated using the conventional Histogram solution with 30 samples with those estimated using the Weibull signal model with 20 samples reveals that the latter is also better than the former, thereby demonstrating that the Weibull-based solution is more efficient than conventional Histogram solution. Furthermore, the probability distributions estimated with the Weibull-based solutions are significantly better than those obtained from the conventional Histogram solution. Thus, from this experiment, the fingerprint database with the Weibull signal model can improve the efficiency and accuracy of the algorithm.

**Figure 6:**
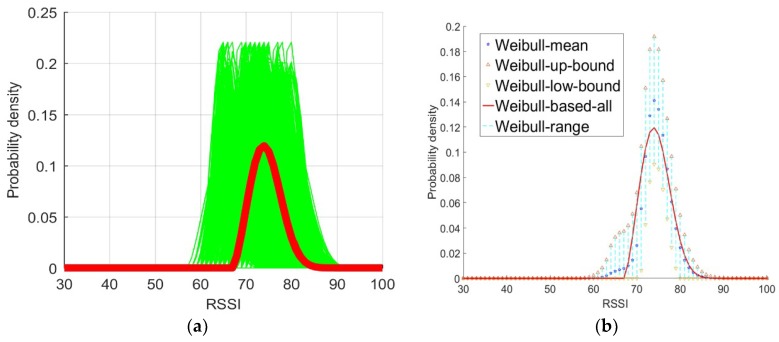
(**a**) Probability densities estimated using all samples (red line) and each probability density function (PDF) estimated with the Weibull signal model for all sessions with sets of 30 RSSI measurement samples (cluster of green lines). (**b**) The cyan dashed line connects the mean of the value of all sessions plus the variance of the value of all sessions (magenta triangles), the mean of the value of all sessions (blue stars), and the mean of the value of all sessions minus the variance of the value of all sessions (inverted magenta triangles); the red line is the baseline distribution.

As shown in Figure 6, every set of 30 RSSI samples was taken from the entire sample data set as one session to estimate the parameters of the Weibull signal model (green line in Figure 6a) for the purpose of comparing the resulting probability density with the baseline probability density derived with all samples (red line in Figure 6a). For a more detailed test, the Weibull signal model was used to derive the probability densities and calculate the mean (blue star in Figure 6b) and variance of all sessions. We obtained the ranges of the variables for all sessions where the variable for each session ranges from the mean plus the variance (magenta triangles in Figure 6b) to the mean minus the variance (inverted magenta triangles in Figure 6b). The estimated Weibull signal model with all samples was used to compute the probability density (shown as the red line in Figure 6b). The results intuitively illustrate that the probability density derived with every set of 30 samples was close to the probability density derived with all samples.

It is not difficult to see that the shapes of most Weibull signal model based probability distributions based on the Weibull signal model derived from 30 RSSI samples were close to that of the baseline distribution (the red line in Figure 6). Hence, according to the test, the Weibull signal model based probability distribution derived from 30 RSSI measurement samples effectively approximated the baseline probability distribution.

### 4.3. Indoor Positioning Performance Evaluation

First, we used all data sets to verify which RSSI range constitutes the best bin for the Weibull PDF algorithm proposed in this paper. Based on the findings shown in Table 1 and Figure 2, we chose ±5 dBm as the RSSI dynamic bin of the Weibull PDF algorithm. In addition, we used three algorithms to calculate the positioning coordinates of all data sets and compared them with the true coordinates recorded to calculate the average error and RMS error of each algorithm. We also plotted the error distribution graph of three algorithms and the probability CDF of the positioning error.

In this study, two dynamic experiments were conducted in which 215 and 100 sets of data were acquired on the second and fourth floors, respectively, within the office building of our laboratory. The test data sets were employed to estimate the position using the histogram algorithm and the two Weibull-based algorithms. All algorithms perform slightly better on the fourth floor than on the second floor. From the actual maps of the two floors (Figure 3 and Figure 4), the result of this experiment could be inferred because the environment on the fourth floor is more open, and the Wi-Fi signal is less disturbed.

The test data sets were applied to estimate the position using the histogram and Weibull-based fingerprint databases. The histogram fingerprint database was generated using Equation (3) with 30 RSSI samples, while the Weibull-based solution was derived from Equation (12) with 20 out of 30 RSSI samples. The experimental results are presented in Table 2 and Table 3. Evidently, the Weibull-based solution performed significantly better than the histogram solution. For a more detailed analysis, the same data sets were utilized to estimate the position using the histogram and Weibull PDF fingerprint databases. The Weibull PDF algorithm clearly performed significantly better than both the conventional histogram algorithm and the Weibull bin algorithm. The accuracy of the Weibull PDF algorithm solution for the data from the second floor was 0.56 m better than that of the histogram solution for the same data set. For the data from the fourth floor, the error of the Weibull PDF algorithm was 0.59 m lower than that of the histogram solution. Compared with the conventional histogram algorithm, the RMS errors of the Weibull PDF algorithm were 20.8% and 35.2% higher in the two different scenarios.

**Figure 7:**
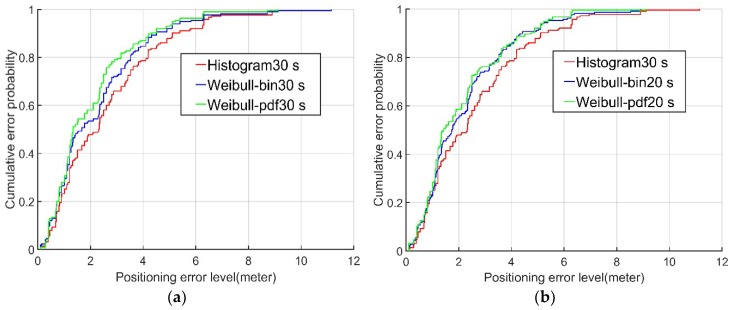
The (**a**,**b**) CDFs of the three algorithms on the second floor.

**Figure 8:**
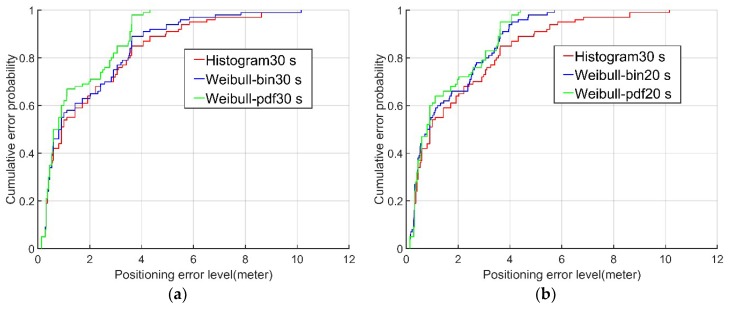
The (**a**,**b**) CDFs of the three algorithms on the fourth floor.

Examining the cumulative probability distributions in the error graph, the positioning results for the data from the second and fourth floors using the Weibull PDF algorithm (green lines in Figure 7 and Figure 8) were 60% and 70%, respectively, when the positioning error was less than 2 m. In contrast, the corresponding results using the conventional histogram algorithm (red lines in Figure 7 and Figure 8) and the Weibull bin algorithm (blue lines in Figure 7 and Figure 8) were only approximately 50% and 60%, respectively, when the positioning error was less than 2 m. When the cumulative probability of the positioning error was 95%, the positioning errors of the Weibull PDF algorithm were 5.22 m and 3.63 m on the second and fourth floors, respectively, while the positioning errors of the conventional histogram algorithm were only 6.3 m and 6.18 m, respectively, and those of the Weibull bin algorithm were 5.81m and 5.45 m, respectively. It is obvious from this that the Weibull PDF algorithm proposed in this paper had the highest accuracy. Even when the established fingerprint database contained only 20 RSSI samples, the Weibull PDF algorithm performed better than the conventional histogram algorithm with 30 RSSI samples.

In general, the three algorithms utilized in the experiment exhibited some points with larger errors, although the Weibull PDF algorithm always performed significantly better than the conventional histogram algorithm and Weibull bin algorithm. The mean errors of the proposed algorithm were 2.03 m and 1.37 m for the two actual scenes on the second and fourth floors, respectively, and 90% and 98% of the positioning errors were within 4 m. In addition, compared with the conventional histogram algorithm, the RMS errors of the Weibull PDF algorithm were 20.8% and 35.2% higher in the two different scenarios. These findings reveal that the proposed algorithm improved not only the positioning accuracy but also the acquisition and calculation efficiency of the fingerprint database.

## 5. Conclusions

The utility of radio-signal-based indoor positioning has recently received increased attention from researchers. Accordingly, in this paper, a method is proposed based on the Bayesian fingerprinting positioning method with Wi-Fi RSSI observables that optimizes the radio map learning and position inference phase to enhance its usability. During the radio map learning phase, the proposed method uses a Weibull–Bayesian density model to represent the PDF of Wi-Fi RSSI observables, which can be calculated with fewer samples. Moreover, the proposed method can calculate the PDF with a higher accuracy than the traditional histogram method. The parameterized Weibull model can greatly reduce both the amount of necessary fieldwork and the cost of the radio map learning phase. Furthermore, the method proposed herein effectively resolves the contradiction between large sampling statistics and data collection efficiency. During the position inference phase, the proposed method calculates the posterior probability using the Bayesian density model and a dynamically defined run-time bin according to real-time RSSI observables rather than the probability distribution of predefined RSSI bins as is accomplished in traditional methods. When implemented on an Android smartphone in different indoor environments, the proposed method enhanced the usability of Wi-Fi Bayesian fingerprinting positioning by requiring a smaller number (i.e., one-third) of signal observables and improved the positioning accuracy by 19–32% in different building environments compared with the classical histogram-based method. In general, the new method proposed in this paper exhibits good prospects.

However, the presented results are only for Wi-Fi fingerprinting positioning, and the proposed method has not been integrated with pedestrian dead reckoning (PDR) or other localization sources; hence, the positioning accuracy is not fully up-to-date. In the future, we will conduct additional studies on multisource integrated positioning. For instance, the fusion of Wi-Fi fingerprinting with PDR and maps will result in a better positioning accuracy.

## Figures and Tables

**Table 1:** Comparison of errors in different received signal strength indication (RSSI) ranges.

	±4 dBm	±5 dBm	±6 dBm
RMS	2.41	2.38	2.38
Mean error (m)	1.85	1.81	1.81
95% error (m)	5.17	4.5	4.69

**Table 2:** Comparison of errors in the three algorithms on the second floor.

	Histogram 30 s	Weibull bin		Weibull PDF	
		20 s	30 s	20 s	30 s
RMS	3.27	2.79	2.9	2.65	2.59
Mean error (m)	2.59	2.21	2.25	2.1	2.03
95% error (m)	6.3	5.39	5.81	5.39	5.22

**Table 3:** Comparison of errors in the three algorithms on the fourth floor.

	Histogram 30 s	Weibull bin		Weibull PDF	
		20 s	30 s	20 s	30 s
RMS	2.87	2.12	2.61	1.94	1.86
Mean error (m)	1.96	1.54	1.79	1.42	1.37
95% error (m)	6.18	4.19	5.45	3.84	3.63
